# Voltage-Gated Sodium Channel (*Vgsc*) Mutation-Based Pyrethroid Resistance in *Aedes aegypti* Populations of Three Endemic Dengue Risk Areas of Sri Lanka

**DOI:** 10.1155/2021/8874092

**Published:** 2021-05-22

**Authors:** Tharaka Ranathunge, Lahiru Udayanga, Sumudu Sarasija, Samudra Karunathilaka, Shavindhya Nawarathne, Haruthra Rathnarajah, Fathima Fazla Dulficar, Fathima Nafla Shafi, Ranil S. Dassanayake, Y. I. Nilmini Silva Gunawardene

**Affiliations:** ^1^Molecular Medicine Unit, Faculty of Medicine, University of Kelaniya, Sri Lanka; ^2^Department of Bio-Systems Engineering, Faculty of Agriculture and Plantation Management, Wayamba University, Sri Lanka; ^3^Department of Chemistry, Faculty of Science, University of Colombo, Sri Lanka; ^4^Business Management School (BMS), Colombo, Sri Lanka

## Abstract

**Background:**

Pyrethroid insecticides are widely used in many countries for chemical-based control of *Ae. aegypti*. Regardless of their efficacy, the constant use of insecticides has induced insecticide resistance mechanisms, such as knockdown resistance (*kdr*) in mosquitoes. Sri Lankan Vector Controlling Entities (VCE) have been using a variety of pyrethroid insecticides as the primary approach for dengue control. However, development of any resistance among the *Aedes* mosquitoes has been limitedly studied in the country. Therefore, the current study was conducted to evaluate the prevalence of F1534C, V1016G, and S989P mutations among *Ae. aegypti* mosquito populations in three dengue endemic high-risk regions of Sri Lanka. *Methodology*. Immature (both pupae and larvae) stages of *Ae. aegypti* mosquitoes were collected from Colombo, Gampaha, and Kandy districts of Sri Lanka from February 2018 to December 2019. Polymerase Chain Reaction- (PCR-) based assay for molecular genotyping of mutations was performed to identify the prevalence of *kdr* mutations in collected *Ae. aegypti* populations, separately. The frequencies of the resistant and susceptible kdr alleles were determined by using the Hardy–Weinberg equilibrium.

**Results:**

The *Ae. aegypti* populations from Colombo, Gampaha, and Kandy districts showed 46%, 42%, and 22% of F1534C mutation allele frequencies, along with 15%, 12%, and 6% of V1016G mutation allele frequencies, respectively. The mutation allele frequencies of S989 in Colombo, Gampaha, and Kandy districts were 9.5%, 8.5%, and 4.5%, respectively. The wild-type (PP) genotype remained predominant within all the three districts, whereas the homogenous (QQ) mutation genotype occurred only in minority. The abundance of Q allele frequency in *Ae. aegypti* mosquitoes was relatively higher for all the three mutations in Colombo.

**Conclusions:**

The findings clearly indicate that long-term insecticide applications and multiple use of pyrethroids have led to the acquisition of *kdr* mutations, leading to the development of insecticide resistance among local *Ae. aegypti* populations, especially in the Colombo and Gampaha districts. Therefore, evaluation of the prevalence levels of these *kdr* mutations highlights the necessity for shifting towards novel vector control strategies.

## 1. Background

The evolution and development of human civilizations on Earth have resulted in encroaching of native habitats of many species by humans. As a result, certain insects that breed in human-occupied regions have coevolved with humans, acquiring affinity towards human blood as their dietary requirements [1]. These blood-requiring insects are disastrous as they can cause or spread many dreadful diseases worldwide. Among these, dengue is considered as one of the most important arboviral diseases at present [2]. At the global level, dengue has threatened approximately 3.9 billion people residing in more than 128 countries [3]. Sri Lanka has been engaged in a fight against dengue, since the mid-1960s, and the disease has become the most severe health burden of the country at present [2]. The worst epidemic of dengue in Sri Lanka was witnessed in 2017 resulting in 186,101 dengue cases along with more than 300 deaths. In 2019, a total of 105,049 dengue cases were reported as the second highest dengue epidemic faced by the country. Meanwhile, a total of 31,139 dengue cases were reported in 2020 [4].

With the absence of vaccines, patient management and vector control are crucial to manage dengue outbreaks in many countries, including Sri Lanka [5, 6]. Therefore, maintaining the *Aedes* vector population, that could cause dengue, chikungunya, yellow fever, and Zika, under the threshold level is one of the key preventive approaches against vector-borne diseases [7]. The current vector control strategies in Sri Lanka mainly focus on environmental management [8], biological control [9–11], and chemical vector control [12] for the suppression of *Aedes* populations, below the threshold levels. Among these, application of larvicides remains as the most widely used chemical control method.

Sri Lankan Vector Controlling Entities (VCE) rely upon a variety of pyrethroids, namely, prallethrin, etofenprox, pyrethrins, permethrin, resmethrin, and sumithrin, as the primary larvicides and adulticides for *Aedes* vector control [13]. However, extensive use of chemical-based vector control methods is known to cause numerous harmful impacts such as the development of insecticide resistance in mosquitoes, unintended side effects on human health or other nontarget populations, and biological magnification [14, 15].

Toxic effects of pyrethroids are mediated through preventing the closure of the voltage-gated sodium channels in the axonal membranes [16, 17]. Extensive usage of pyrethroids to control *Ae. aegypti*, the primary vector of dengue, could lead to the selection of insecticide resistance-related mutations in mosquitoes [18]. In insects, the “knockdown resistance” (*kdr*) is one of the most widespread mechanisms that could cause resistance to pyrethroids and Dichloro-Diphenyl-Trichloroethane (DDT) [19]. The *kdr* resistance occurs due to mutations in the voltage-sensitive sodium channel (*Vssc*) or voltage-gated sodium channel (*Vgsc*), coded by the *Vssc* gene. The foremost function of *Vgsc* is the initiation and transmission of action potentials in neuron cells [20]. Due to mutations in the *Vssc* gene, insecticides are unable to bind and block the function of *Vgsc*, resulting in resistance [21].

Around eleven mutations linked to pyrethroid resistance in *Ae. aegypti* mosquitoes have been identified at the global level. Among them, five mutations, namely, S989P, I1011M, V1016G, F1534C, and V410L, have been functionally confirmed to confer pyrethroid resistance [22]. Only three *kdr* mutations, the V1016G, S989P, and F1534C, have been recognized as commonly occurring amino acid substitutions among *Aedes* mosquitoes in the Southeast Asian region [23]. The F1534C mutation, which reduces permethrin binding to *Vgsc* channels, has been reported as the most common *kdr* mutation in the Southeast Asia [22].

The development of *kdr* mutation in *Ae. aegypti* populations could significantly reduce the efficacy of chemical-based vector control approaches in Sri Lanka. This will cause a direct impact on the success of dengue epidemic management in the country, leading to incidence of more severe dengue epidemics. In a recent study, Fernando et al. [24] have reported the presence of V1016G and S989P mutant alleles in the *Vgsc* of Sri Lankan *Ae. aegypti* populations for the first time. A study conducted by Endersby-Harshman et al. [25] to investigate the geographical distributions of three sodium channel mutations found in *Ae. aegypti* from the Indo-Pacific region has also presented evidence for existence of these mutations in Sri Lanka, which may have been attained by *Ae. aegypti* via genetic invasion. However, more detailed studies on this aspect are lacking in Sri Lanka. Therefore, the current study was conducted to determine the spatial distribution pattern of V1016G, F1534C, and S989P *kdr* mutations, among *Ae. aegypti* mosquito populations in three different high-risk geographical regions for dengue in Sri Lanka.

## 2. Methodology

### 2.1. Study Area

Over the past few years, the highest dengue burden in Sri Lanka has occurred in the districts of Colombo, Gampaha, and Kandy. The district of Colombo is the commercial capital of Sri Lanka (6.70°N and 80.22°E). Being located in the lowland of the country, Colombo is the most urbanized metropolitan area of the country. In 2019, 13.7% (*n* = 4257) of 31,139 dengue cases were observed in Colombo [4] as the most high-risk area for dengue. Meanwhile, Gampaha district (7°05′N and 79°59′E) reported the third highest number of dengue cases as 2655 (8.5%) [4]. Both Colombo and Gampaha districts are located in the low country wet zone of Sri Lanka. Meanwhile, Kandy district (6.93° to 7.50°N and 81.04°E) is located in the central highlands, covering an area of 1940 km^2^ with a wide array of natural environmental features in contrast to Colombo. It is of major tourist interest, due to its natural locations and places of historical and religious importance. At present, Kandy district remains as the second high-risk area for dengue transmission in the country, reporting 11.0% (*n* = 3432) of the dengue cases reported in 2019 [4]. Due to the severe dengue risk, these three districts are characterized by a higher level of insecticide applications to control *Aedes* vectors. Therefore, the districts of Colombo, Gampaha, and Kandy were selected as the study areas in this study, which is important for evidence-based vector control.

### 2.2. Collection of *Ae. aegypti* Mosquitoes

Immature (both pupae and larvae) stages of mosquitoes were collected from each district using ovitraps from February 2018 to December 2019. The collected samples were transported to the entomology laboratory at the Molecular Medicine Unit, Faculty of Medicine, University of Kelaniya, Sri Lanka.

### 2.3. Identification of Field-Caught Mosquitoes

Larvae were placed individually on a depression microscopic slide with a minimum amount of water and were identified under a light microscope (Olympus Optical Co. Ltd., Tokyo) with an objective lens (×10). Stages III and IV instar larvae of *Ae. aegypti* collected from the field were directly identified using morphological keys [26], while stages I and II were reared to stage III prior to identification. Collected pupae were reared until the adult stage, and adult mosquitoes were identified by an achromatic magnification lens (×10) using standard morphological keys prepared for *Aedes* mosquitoes [26].

### 2.4. DNA Extraction and *kdr* Mutation Genotyping for *Ae. aegypti*

A total of 974 *Ae. aegypti* larvae and adults (reared from pupal stage) collected from all three districts were subjected to DNA extraction using QIAamp DNA Mini Kit according to the manufacturer's instructions [27]. The extracted DNA was preserved at -20°C, until detection of different mutations.

### 2.5. Detection of F1534C and Genotyping

To detect the F1534C mutation, allele-specific PCR (AS-PCR) was performed as follows [24]. Extracted DNA was amplified in a 25 *μ*l of PCR mixture containing 1x PCR buffer, MgCl_2_ (1.5 mM), dNTPs (200 *μ*M), forward primer (0.5 *μ*M), reverse primer (Cys1534; 0.165 *μ*M), common reverse primer (Cpr; 0.5 *μ*M), Taq DNA polymerase enzyme (0.2 U), and template DNA (2 ng). Cycling conditions were hot started at 95°C for 2 min followed by 35 cycles, each of denaturation at 95°C for 30 s, annealing at 60°C for 30 s, and extension at 72°C for 30 s, followed by the final extension at 72°C for 2 min. Subsequently, 10 *μ*l of amplified products was loaded with 2 *μ*l of 5x gel loading dye in 0.5x TBE buffer, along with a 50 bp DNA marker. Finally, PCR products were visualized by gel electrophoresis (Cell Biosciences, USA) on a 1.5% agarose gel at 150 V for 30 min [24].

### 2.6. Detection of V1016G and Genotyping

Allele-specific PCR (AS-PCR) was performed to detect the V1016G mutation in *Ae. aegypti* [24] by amplifying the extracted DNA in a 25 *μ*l of PCR mixture containing 1x PCR buffer, MgCl_2_ (1.5 Mm), dNTPs (200 *μ*M), forward primer (V1016G; 0.25 *μ*M), reverse primer (0.125 *μ*M) specific for either glycine (Gly1016R) or valine (Val1016R), Taq DNA polymerase enzyme (0.2 U), and template DNA (2 ng). Cycling conditions were hot started at 95°C for 2 min followed by 35 cycles each of denaturation at 95°C for 30 s, annealing at 60°C for 30 s, and extension at 72°C for 30 s, followed by the final extension at 72°C for 2 min. Subsequently, 10 *μ*l of amplified products was loaded with 2 *μ*l of 5x gel loading dye in 0.5x TBE buffer along with a 50 bp DNA marker. Finally, PCR products were visualized by gel electrophoresis (Cell Biosciences, USA) on a 1.5% agarose gel at 150 V for 30 min [24].

### 2.7. Detection of S989P and Genotyping

A similar PCR (AS-PCR) was performed to detect the S989P mutation by amplifying extracted DNA in a 25 *μ*l of PCR mixture containing 1x PCR buffer, MgCl_2_ (1.5 Mm), dNTPs (200 *μ*M), forward primer (0.5 *μ*M), reverse primer (0.5 *μ*M), Taq DNA polymerase enzyme (0.2 U), and template DNA (2 ng) [28]. Cycling conditions were hot started at 95°C for 2 min followed by 35 cycles each of denaturation at 95°C for 30 s, annealing at 63°C for 30 s, and extension at 72°C for 30 s, followed by the final extension at 72°C for 2 min. Subsequently, 10 *μ*l of amplified products was loaded with 2 *μ*l of 5x gel loading dye in 0.5x TBE buffer along with a 50 bp DNA marker. Finally, PCR products were visualized by gel electrophoresis (Cell Biosciences, USA) on a 1.5% agarose gel at 150 V for 30 min [24].

### 2.8. Data Analysis

The frequencies of the resistant (Q) and susceptible (P) *kdr* alleles for each of the point mutation were determined by the Hardy–Weinberg Equilibrium using an online calculator [29]. The mean P and Q allele frequencies of *Ae. aegypti* mosquitoes from three study areas in 2018 and 2019 were square root transformed. The Bray-Curtis similarity-based Distance-Based Redundancy Analysis (dbRDA) was performed to highlight and visually represent the underlying segregation patterns of the *Ae. aegypti* populations from three districts, based on the overall variations in the frequencies of the resistant (Q) and susceptible (P) *kdr* alleles of F1534C, V1016G, and S989P point mutations. The Plymouth Routines in Multivariate Ecological Research (PRIMER version 6) was used for statistical analysis.

## 3. Results

**Figure 1:**
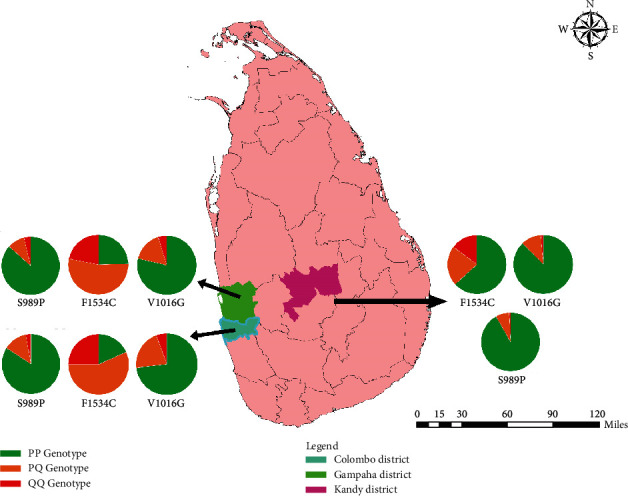
Spatial distribution of genotypes corresponding to F1534C and V1016G mutations in Sri Lankan *Ae. aegypti*.

**Figure 2:**
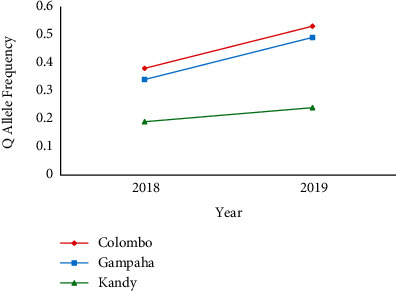
Mutation (Q) allele frequency of F1534C mutation in Sri Lankan *Ae. aegypti* in 2018 to 2019.

A total of 974 *Ae. aegypti* mosquitoes caught from the wild were screened for *Vgsc* mutations as depicted in Figure 1. In the F1534C genotyping, the PQ genotype dominated among the mosquito samples of Colombo (43.7%), while PP genotype remained dominant among *Ae. aegypti* mosquitoes from Gampaha (44.5%) and Kandy (71.7%) in 2018 as shown in Table 1. However, the PQ genotype denoted a notable increment in 2019 at the position 1534 in domain III of the *Vgsc* gene in *Ae. aegypti* mosquitoes from all the three districts, when compared to 2018. In fact, the PQ genotype was dominant in both Colombo and Gampaha districts accounting for 56.8% and 53.6% frequencies, respectively, among the screened *Ae. aegypti* mosquitoes in 2019. When the allele frequencies are considered, it was evident that Q allele frequency (mutation) accounted for around one-third (33.3%) of the screened *Ae. aegypti* mosquito populations from Colombo and Gampaha in 2018. Further, the Q allele frequency denoted gradual increments in 2019 within the mosquitoes from all study districts (Figure 2).

**Figure 3:**
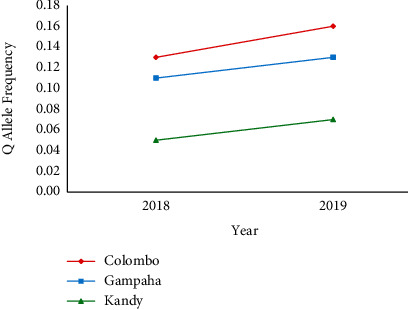
Mutation (Q) allele frequency of V1016G mutation in Sri Lankan *Ae. aegypti* in 2018 to 2019.

The PP genotype remained predominant at position 1016 in domain II, segment 6, of the *Vgsc* gene in *Ae. aegypti* mosquito for the V1016G mutations in *Ae. aegypti* mosquito populations, while the highest frequency of the QQ genotype was observed in Colombo as 5.1% and 5.7% in 2018 and 2019, respectively. In case of allele frequency, P allele was predominant in the mosquito populations from all three districts. However, Q allele was relatively more abundant among the *Ae. aegypti* mosquitoes of Colombo, followed by Gampaha (Table 2). In general, mosquitoes from all the three districts denoted a gradually increasing trend of the Q allele frequency in 2019 than in 2018, for the V1016G mutation (Figure 3).

**Figure 4:**
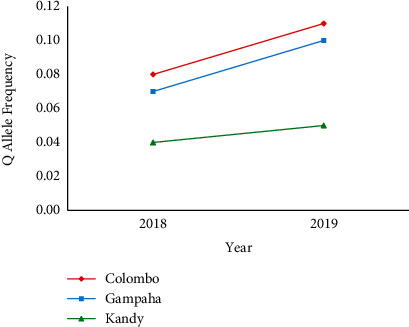
Mutation (Q) allele frequency of S989P mutation in Sri Lankan *Ae. Aegypti* in 2018 to 2019.

In the positions 1016 and 989 for the S989P mutations, the PP genotype dominated (>80%) in all the *Ae. aegypti* mosquito populations. However, the homozygous resistance genotype was relatively higher in the *Ae. aegypti* population from the district of Colombo, denoting 1.9% and 3.6% in 2018. Interestingly, *Ae. aegypti* mosquitoes from Gampaha accounted for the highest QQ genotypic frequency as 4.5% in 2019 (Table 3). In case of allele frequency, similar to other mutations, P allele was predominant in the mosquito populations from all three districts. However, *Ae. aegypti* mosquitoes from Colombo, Gampaha, and Kandy districts denoted a gradually increasing trend of the Q allele frequency in 2019 than in 2018 (Figure 4).

### 3.1. Spatial Dissimilarities in the Mutation Abundance among *Ae. aegypti* Mosquito Populations

**Figure 5:**
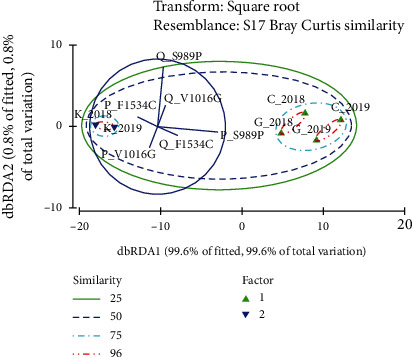
Clustering of *Ae. aegypti* populations from studied districts based on allele frequency of F1534C and V1016G mutations in Sri Lanka.

The clustering status of *Ae. aegypti* populations from studied districts, based on allele frequencies of F1534C, V1016G, and S989P mutations corresponding to 2018 and 2019, is depicted in Figure 5. The two dbRDA axes cumulatively accounted for 100% of the overall variations in the dataset, suggesting a perfect fit of the analysis. The dbRDA axis 1 corresponded for 99.6% of the variation becoming the dominating axis. Q allele frequency of F1534C (85.9%), S989P (65.4%), and V1016G (38.2%) mutations was the most significant loading variables that positively contributed to the dbRDA axis 1. Meanwhile, P allele frequency of F1534C (58%) and V1016G (34.5%) contributed negatively. In case of dbRDA axis 2, Q allele frequency of S989P (73.5%) and F1534C (24.1%) contributed positively, while P allele frequency of F1534C (34.8%) and V1016G (5.6%) contributed negatively, as significant loading variables.

*Ae. aegypti* populations of Colombo and Gampaha in 2018 formed a subcluster sharing a similarity of 97.4% for P and Q allele frequencies of the three-point mutations. Meanwhile, *Ae. aegypti* mosquitoes from Colombo and Gampaha of 2019 clustered together, while *Ae. aegypti* mosquitoes collected in 2018 and 2019 from Kandy formed another two subclusters at the similarity level of 96.6% (Figure 5). Interestingly, *Ae. aegypti* populations of Colombo and Gampaha districts formed a major cluster, denoting a similarity level of 95.3% in overall P and Q allele frequencies. As depicted in Figure 5, the *Ae. aegypti* populations of Colombo and Gampaha districts denoted a 25.3% level of dissimilarity from the *Ae. aegypti* population of Kandy. Based on the loading coefficients, it appears that the relatively higher Q allele frequencies of F1534C, V1016G, and S989P in the *Ae. aegypti* populations of Colombo and Gampaha districts are the cause for formation of a major cluster at a 95.3% similarity level. Since the Q allele frequencies in the *Ae. aegypti* populations of Kandy are notably low, it has formed a separate major cluster sharing only 74.6% of similarity (Figure 5).

### 3.2. Complete Genotype Distribution

Among the calculated complete genotypes, the FF VV SS dominated (>34%) in all the *Ae. aegypti* populations in 2018 from all the three districts. Meanwhile, FC VV SS also showed a relatively higher abundance in the districts of Colombo and Gampaha in 2018. In 2019, the relative abundance of FC VV SS showed an increment among the *Ae. aegypti* from all the three districts (Table 4). Interestingly, the FF VV SS remained prominent in the *Ae. aegypti* populations from Kandy in 2018 (71.7%) and 2019 (64.1%). On the contrary, the homozygous resistance genotypes CC GG PP were relatively low (<3.5%) in the *Ae. aegypti* populations from the district of Colombo, Gampaha, and Kandy in 2018 and 2019 (Table 4).

## 4. Discussion

Despite the continuous application of larvicides and adulticides, severe incidence of dengue epidemics reported from Sri Lanka strongly suggests the possibility of insecticide resistance among *Aedes* vectors. The present study was undertaken to further investigate the presence of F1534C, V1016G, and S989P *kdr* mutations, among *Ae. aegypti* populations in different geographical regions (Colombo, Gampaha, and Kandy) of Sri Lanka and their development during two preceding years (2018 and 2019).

According to the current study, all the three mutations, F1534C, V1016G, and S989P, were detected among the *Ae. aegypti* populations in Colombo, Gampaha, and Kandy districts from 2018 to 2019. A study conducted by Endersby-Harshman et al. has reported five allelic combinations to be common in *Ae. aegypti* mosquitoes from the Indo-Pacific region [25]. Among them, F1534C, V1016G, and S989P mutations have been recognized as the most commonly found *Vgsc* mutations in pyrethroid-resistant *Ae. aegypti* in many Southeast Asian countries such as Cambodia [30], Indonesia [31, 32], Laos [33], Malaysia [34], Myanmar [35], Singapore [36], Thailand [37], and Vietnam [38]. In addition to Southeast Asian countries, high prevalence levels of pyrethroid-resistant mutations have been reported in many countries worldwide due to overapplication of pyrethroids [39, 40]. In the current study, both Colombo and Gampaha districts remain as urbanized areas with relatively higher risk for dengue incidence. Therefore, the vector control activities are more intensive in these two districts leading to elevated chances for pyrethroid resistance development. Findings of the current study are also supported by Fernando et al., who have reported the presence of F1534C, V1016G, and S989P mutant alleles in the *Vgsc* genes of Sri Lankan *Ae. aegypti* [24].

Among the three point mutations, mutant alleles of F1534C were the most predominant, denoting a relatively extensive dispersion across all of the study sites. High prevalence rates of V1016I and F1534C have been reported in Latin America, including Brazil [41], Venezuela [42], Colombia [43], and Jamaica [44]. In Mexico, a study conducted by Aponte et al. [39] has also reported the widespread distribution of F1534C and I1016 mutations and suggested that higher F1534C frequency is more likely to allow other mutations to evolve. Similarly, in the current study, the Colombo district, which had a higher F1534C frequency, showed the presence of V1016G and S989P mutations at relatively higher levels.

A simple AS-PCR technique has been successfully applied in the current study to detect F1534C, V1016G, and S989P in *Ae. aegypti* populations of Sri Lanka. Genotyping of F1534C exhibited that the heterozygous (PQ) genotype was slightly higher than the homozygous susceptible gene (PP) in both Colombo and Gampaha districts. The frequencies of mutant alleles of all F1534C, V1016G, and S989P point mutations denoted a gradual increment from 2018 to 2019 in *Ae. aegypti* populations, showing that those mutant alleles are currently been selected in *Ae. aegypti* populations in Sri Lanka. Several previous studies have shown that the homozygous mutant individuals would be more resistant, while wild-type homozygous would be more susceptible. Further, an intermediate resistance would be displayed by heterozygous mosquitoes to pyrethroids [18]. The intermediate resistance of heterozygous mosquitoes clearly suggests that mutation is not the only mechanism involved and that either other *kdr* or enzymatic mechanisms may confer cross resistance or enhance the resistance. The cooccurrence of two or three *kdr* mutations in *Ae. aegypti* has been reported in many countries and is believed to cause a higher level of resistance to pyrethroids [39–42]. Further, it is reported that the F1534C mutation could potentially compensate for any reduction in fitness caused by the V1016G and S989P mutations [45].

According to Brengues et al. [19], knockdown resistance against pyrethroids in *Ae. aegypti* may possibly be conferred by one or more point mutations in the Vgsc locus. For instance, Amelia-Yap et al. [46] have reported the cooccurrence of V1016G/S989P point mutations as a common cooccurrence pattern in tested pyrethroid-resistant *Ae. aegypti* populations. Hence, the increment of the insensitivity of *Vgsc* to pyrethroids could be experienced as a result of this phenomenon. Although the S989P mutation was detected, it cannot be considered as the main cause for the insecticide resistance, since the S989P mutation has always been associated with the V1016G mutation, until the S989P mutation occurs alone [34, 35]. However, according to a study conducted in Thailand, the V1016G mutation can stand alone despite the absence of the S989P mutation in *Ae. aegypti* [45]. Hence, it can be extrapolated that the intensifying effect of pyrethroid resistance may have been resulted from the synergized S989P mutation.

Although Du et al. [21] have further proved that the S989P mutation is not having any additive effect to the V1016G mutation, a recent contradictory study has highlighted that the existence of the S989P mutation can highly diminish the sensitivity and the susceptibility of *Aedes* vectors to pyrethroids [47]. A recent study conducted in Indonesia has also emphasized the cooccurrence of the V1016G and S989P mutations in high frequencies among the Asian *Ae. aegypti* populations [31, 32]. In the current study, only *Ae. aegypti* mosquito populations from the districts of Colombo and Gampaha, that had relatively higher resistant gene frequencies of V1016G mutation, were also having S989P mutations at relatively notable levels. However, it is still debatable if the S989P mutation was responsible for causing the additive role in pyrethroid resistance development in *Ae. aegypti*. This signifies the importance of further in-depth studies for the novel discoveries related to this aspect.

The presence of F1534C, V1016G, and S989P *kdr* mutations in *Ae. aegypti* mosquito populations from different regions in Sri Lanka indicates the prevalence of insecticide resistance. It is obvious that the findings of the current study are based on only 974 *Ae. aegypti* mosquito larvae, which is limited. However, the regions selected for the study (Colombo, Gampaha, and Kandy) had been treated with large amounts of insecticide for a long time, compared with other areas of the country. Therefore, the extensive use of pyrethroids to control dengue should be the cause for this development of pyrethroid resistance in *Aedes* mosquitoes [48]. Furthermore, the presence of mutated mosquitoes even within a low sample size in the current study predicts that there is a high likelihood of having a notable degree of resistant *Ae. aegypti* mosquitoes in the wild. If the current rate of pyrethroid use persists in Sri Lanka, prevalence and distribution of *kdr* mutations could rapidly elevate, challenging the successful management of dengue epidemics in Sri Lanka, through conventional chemical-based control. Although the current study focused on the allele frequency as single mutations, the cooccurrence of the three mutations among the *Ae. aegypti* mosquitoes was not investigated, due to limitations in funds. Such study would reveal the strength of the resistance to pyrethroid insecticides [21], which is of higher significance. Therefore, in-depth studies are recommended to further investigate the combined effect of the alleles in inducing pyrethroid resistance in *Ae. aegypti*.

The future holds promising prospects in development of efficient insecticides and new mosquito control strategies. However, their inclusive implementation in tropical regions will require a decade at minimum [49, 50]. Thus, characterizing molecular mechanisms that may trigger insecticide resistance in *Aedes* mosquitoes is vital for tracking down resistance alleles and enhancing resistance management strategies [51]. Therefore, further identification of *kdr* mutations, that cause insecticide resistance, is crucial in the country. The present study has revealed the presence of insecticide resistance among the wild *Ae. aegypti* populations through target site resistance mechanisms. Such mutations could exert negative fitness costs influencing the evolution of further insecticide resistance in field populations of mosquitoes. Therefore, it is crucial that such impacts are properly understood and taken into consideration, when designing and implementing future insecticide resistance management strategies. In general, the detection of *kdr* mutations in *Ae. aegypti* populations could enable the monitoring of insecticide resistance occurrence and guiding of vector control approaches in Sri Lanka. In addition, the society needs to progressively upgrade their lifestyle to allow better control of *Ae. aegypti* effectively.

## 5. Conclusion

According to the current study, all the three mutations, F1534C, V1016G, and S989P, were detected among the *Ae. aegypti* populations in Colombo, Gampaha, and Kandy districts. Among the three-point mutations, mutant alleles of F1534C were the most predominant, denoting a relatively extensive dispersion across all the study sites. The heterozygous (PQ) genotype was slightly higher than the homozygous susceptible gene (PP) in both Colombo and Gampaha districts. The frequencies of mutant alleles of all F1534C, V1016G, and S989P point mutations denoted a gradual increment from 2018 to 2019 in *Ae. aegypti* populations, showing that mutant alleles are being selected within *Ae. aegypti* populations in Sri Lanka. This could impose a serious threat to the efficacy of current dengue vector control strategies, as VCE in Sri Lanka mainly focus on chemical-based dengue control methods. Therefore, evaluation of the prevalence levels of these *kdr* mutations highlights the necessity for shifting towards novel vector control strategies.

## Figures and Tables

**Table 1:** The F1534C genotyping and frequency of *kdr* alleles in Sri Lankan *Ae. aegypti*.

District	Year	Total number of mosquitoes	Genotype			Allele frequency	
			PP	PQ	QQ	P	Q
Colombo	2018	158	64 (40.5%)	69 (43.7%)	25 (15.8%)	0.62	0.38
	2019	192	35 (18.2%)	109 (56.8%)	48 (25.0%)	0.46	0.53
Gampaha	2018	165	73 (44.5%)	72 (43.9%)	19 (11.6%)	0.66	0.34
	2019	179	44 (24.6%)	96 (53.6%)	39 (21.8%)	0.51	0.49
Kandy	2018	127	91 (71.7%)	25 (19.7%)	11 (8.7%)	0.81	0.19
	2019	153	98 (64.1%)	36 (23.5%)	19 (12.4%)	0.76	0.24

**Table 2:** The V1016G genotyping and frequency of *kdr* alleles in Sri Lankan *Ae. aegypti*.

District	Year	Total number of mosquitoes	Genotype			Allele frequency	
			PP	PQ	QQ	P	Q
Colombo	2018	158	125 (79.1%)	25 (15.8%)	8 (5.1%)	0.87	0.13
	2019	192	143 (74.5%)	38 (19.8%)	11 (5.7%)	0.84	0.16
Gampaha	2018	165	137 (83.0%)	20 (12.1%)	8 (4.8%)	0.89	0.11
	2019	179	141 (78.8%)	29 (16.2%)	9 (5.0%)	0.87	0.13
Kandy	2018	127	113 (88.9%)	14 (11.0%)	00 (0%)	0.95	0.05
	2019	153	133 (86.9%)	17 (11.1%)	3 (1.9%)	0.93	0.07

**Table 3:** The S989P genotyping and frequency of *kdr* alleles in Sri Lankan *Ae. aegypti*.

District	Year	Total number of mosquitoes	Genotype			Allele frequency	
			PP	PQ	QQ	P	Q
Colombo	2018	158	136 (86.1%)	19 (12.0%)	03 (1.9%)	0.92	0.08
	2019	192	158 (82.3%)	27 (14.1%)	07 (3.6%)	0.89	0.11
Gampaha	2018	165	146 (88.5%)	14 (8.5%)	05 (3.0%)	0.93	0.07
	2019	179	152 (84.9%)	19 (10.6%)	08 (4.5%)	0.90	0.10
Kandy	2018	127	117 (92.1%)	10 (7.9%)	00 (0%)	0.94	0.04
	2019	153	140 (91.5%)	11 (7.2%)	02 (1.3%)	0.95	0.05

**Table 4:** Genotype distribution for the voltage-gated sodium channel (Vgsc) mutations in Sri Lankan *Aedes aegypti* populations.

Genotype	Colombo		Gampaha		Kandy	
	Frequency		Frequency		Frequency	
	2018	2019	2018	2019	2018	2019
FF VV SS	55 (34.8%)	35 (18.2%)	74 (44.8%)	40 (22.3%)	91 (71.7%)	98 (64.1%)
FF VV SP	00	00	00	00	00	00
FF VV PP	00	00	00	00	00	00
FF VG SS	9 (5.7%)	00	00	00	00	00
FF VG SP	00	00	00	4 (2.2%)	00	00
FF VG PP	00	00	00	00	00	00
FF GG SS	00	00	00	00	00	00
FF GG SP	00	00	00	00	00	00
FF GG PP	00	00	00	00	00	00
FC VV SS	50 (31.6%)	84 (43.8%)	58 (35.1%)	84 (46.9%)	15 (11.8%)	23 (15.0%)
FC VV SP	3 (1.9%)	00	00	00	00	00
FC VV PP	00	00	00	00	00	00
FC VG SS	5 (3.6%)	12 (6.03%)	6 (3.6%)	00	4 (3.1%)	7 (4.6%)
FC VG SP	11 (7.0%)	13 (6.8%)	8 (4.8%)	12 (6.7%)	6 (4.7%)	6 (3.9%)
FC VG PP	00	00	00	00	00	00
FC GG SS	00	00	00	00	00	00
FC GG SP	00	00	00	00	00	00
FC GG PP	00	00	00	00	00	00
CC VV SS	17 (10.8%)	24 (12.5%)	5 (3.0%)	17 (9.5%)	7 (5.5%)	12 (7.8%)
CC VV SP	00	00	00	00	00	00
CC VV PP	00	00	00	00	00	00
CC VG SS	00	11 (5.7%)	00	11 (6.1%)	00	00
CC VG SP	00	00	6 (3.6%)	00	4 (3.1%)	4 (2.6%)
CC VG PP	00	2 (1.0%)	00	2 (1.1%)	00	00
CC GG SS	00	00	3 (1.8%)	00	00	00
CC GG SP	5 (3.2%)	6 (3.1%)	00	3 (1.7%)	00	1 (0.7%)
CC GG PP	3 (1.9%)	5 (2.6%)	5 (3.0%)	6 (3.4%)	00	2 (1.3%)

## Data Availability

Raw data supporting the findings are available from the authors on request.

## Abbreviations

kdr: Knockdown resistance
VCE: Vector Controlling Entities
WHO: World Health Organization.
